# Chicken Embryos as a Potential New Model for Early Onset Type I Diabetes

**DOI:** 10.1155/2014/354094

**Published:** 2014-07-13

**Authors:** Liheng Shi, Michael L. Ko, Cathy Chia-Yu Huang, So-Young Park, Min-Pyo Hong, Chaodong Wu, Gladys Y.-P. Ko

**Affiliations:** ^1^Department of Veterinary Integrative Biosciences, College of Veterinary Medicine and Biomedical Sciences, Texas A&M University, 4458 TAMU, College Station, TX 77843-4458, USA; ^2^Department of Nutrition, Texas A&M University, College Station, TX 77843-4458, USA; ^3^Texas A&M Institute of Neuroscience, Texas A&M University, College Station, TX 77843-445, USA

## Abstract

Diabetic retinopathy (DR) is the leading cause of blindness among the American working population. The purpose of this study is to establish a new diabetic animal model using a cone-dominant avian species to address the distorted color vision and altered cone pathway responses in prediabetic and early diabetic patients. Chicken embryos were injected with either streptozotocin (STZ), high concentration of glucose (high-glucose), or vehicle at embryonic day 11. Cataracts occurred in varying degrees in both STZ- and high glucose-induced diabetic chick embryos at E18. Streptozotocin-diabetic chicken embryos had decreased levels of blood insulin, glucose transporter 4 (Glut4), and phosphorylated protein kinase B (pAKT). In STZ-injected E20 embryos, the ERG amplitudes of both a- and b-waves were significantly decreased, the implicit time of the a-wave was delayed, while that of the b-wave was significantly increased. Photoreceptors cultured from STZ-injected E18 embryos had a significant decrease in L-type voltage-gated calcium channel (L-VGCC) currents, which was reflected in the decreased level of L-VGCC*α*1D subunit in the STZ-diabetic retinas. Through these independent lines of evidence, STZ-injection was able to induce pathological conditions in the chicken embryonic retina, and it is promising to use chickens as a potential new animal model for type I diabetes.

## 1. Introduction

Diabetes is a fast-growing global problem, and diabetic retinopathy (DR) is the leading cause of blindness among Americans over 40-year old [1–3]. Nearly all patients with type 1 diabetes and more than 60% of those with type 2 diabetes will develop DR [4–6]. Diabetic retinopathy is a dual disorder with microvascular complications and retinal degeneration [7] with a projected prevalence of more than 11 million patients by 2030 in the USA [8]. Historically, DR has been investigated and treated as a complication of retinal vasculature [7]. However, recent developments of highly sensitive techniques, such as multifocal ERG [9] show that the retina starts to degenerate in early diabetes [7, 10] prior to clinical signs of DR and any vascular complications. The distorted color vision and altered cone pathway responses, including the delayed ERG a-wave implicit time, are among the first clinical signs in early stage diabetic patients without DR [11–13], indicating that the cone photoreceptors are compromised in early diabetes [11–13]. In humans, diabetes causes dysfunction [14] and loss of the blue-light sensitive S-cones [11, 14–16]. In hyperglycemic animals with DR, cone photoreceptor degeneration is the predominant dysfunction [7]. Therefore, it is critical to understand how cone photoreceptors are altered under early diabetic states, so that potential therapeutic or preventive treatments could be developed.

There are various DR models from dogs, rodents, to zebrafish [7]. While these animal models have certain characteristic diabetic phenotypes, none are without limitations [7, 17]. It takes 3–5 years to develop DR lesions after induction of diabetes in dogs [17]. Streptozotocin- (STZ-) induced diabetic rats or mice show similar signs of early DR to that of humans. Thus, the rodent models are the most used DR animal models. However, certain biochemical changes, ganglion cell apoptosis, or signs of Müller cell reactivity are inconsistent between STZ-induced diabetic rats and mice [17]. Rodent retinas do not have a macula. As such, they cannot serve as an adequate model of diabetic macular edema that happens in humans [17]. Chronic inflammation associated with diabetes is known to contribute to DR [18], but the genomic responses in mouse models poorly mimic human inflammatory diseases [19]. While one major advantage of using mice in research is for their genetic manipulations, some wild-type mouse strains harbor preexisting abnormal retinas [20]. Furthermore, the rod-dominant nocturnal retinas might not be the best model to address the dysfunction and apoptosis of cones in human DR. Hence, there is a need to develop new diabetic animal models that will complement the current animal models and address certain aspects of human DR.

Chickens have yet to become a widely used animal model for human disease research mainly because they are not mammalian, and their genetic makeup is ~70% homologous to humans. While the methods to produce transgenic birds have been developed, it is still more technically challenging to generate transgenic chickens compared to the making of stable transgenic mouse lines [21]. However, chickens are diurnal with complex color vision, just like humans. Spontaneous mutant blind chickens have been used recently as a model for photoreceptor-degenerative blindness [22]. The fact that the chicken retina is cone-dominant makes it a suitable candidate to study human cone photoreceptor-related degenerative diseases. Also, the abundance of retinal tissue per eye in chickens allows for molecular and biochemical assays without sacrificing or pooling large numbers of animals. While STZ successfully induces diabetes in dogs, rats, and mice, it fails to induce diabetes in adult birds [23], mainly due to species-dependent susceptibility to STZ in the pancreas islets [24]. Interestingly, cultured human islets are also highly resistant to STZ [24, 25]. In order to successfully induce diabetes in chickens, we took advantage of the fact that the pancreas is not fully developed in chicken embryos [26], but cone photoreceptors are functional during the late embryonic stages [27–30]. We injected STZ into the amnion layer* in ovo* and successfully induced type 1 diabetes. Here, we present morphological, physiological, and molecular evidence of our newly established chicken model that is comparable to the existing rodent diabetic retinopathy models. Our new diabetic model using cone-dominant chickens is complementary to the existing rodent models, which will allow researchers to address how early diabetic insults cause cone dysfunction and apoptosis and allow for potential treatments in the future.

## 2. Materials and Methods

### 2.1. Preparation of STZ and High Concentrations of Glucose

A sodium citrate buffer solution (citric buffer; 10 mM, pH 4.0) was prepared to dissolve STZ and served as the vehicle. Streptozotocin (STZ; Enzo Life Sciences; Farmingdale, NY, USA) was prepared freshly and dissolved in the citric buffer at 50 mg/mL. Glucose was dissolved in distilled water at 250 mg/mL as a stock solution and stored at −80°C for up to one month. Prior to injections, the STZ or glucose stock solution was filtered through a 0.22 *µ*m syringe filter. Various doses of STZ or glucose were injected into fertilized eggs based on the weight of the whole egg (STZ, ranging from 250 to 300 mg/kg-egg weight; glucose, 2500 or 3000 mg/kg-egg weight). The volume of each injection was between 250 and 350 *µ*L per egg.

### 2.2. *In Ovo* Injections and Maintenance of Chick Embryos

Fertilized eggs (*Gallus gallus*, Single Comb White Leghorn) were obtained from the Poultry Science Department, Texas A&M University (College Station, TX, USA). All chicken embryos were maintained at 39°C ± 0.5°C. At embryonic day 8 (E8), chick embryos were kept in incubators equipped with lights and timers programmed for 12 : 12 h cyclic light-dark cycles. At E12, the* in ovo* injection procedures were carried out in a sterilized culture hood. Eggs were placed air-sac up and first cleaned with 70% ethanol. A small window (less than 2 cm^2^) on the shell above the air-sac was opened, and the shell membrane was carefully peeled away. The injection needle penetrated through the membrane layers without breaking blood vessels. The vehicle, STZ, or glucose solution (0.25–0.3 mL) was injected into the amnion layer, which was directly outside of the embryos, with a 30G needle during the “light phase” of incubation. After the injection, the small shell opening was covered with two pieces of medical tape to prevent infections. Injected embryos were then returned to the light programmed incubators. At E18–E20, the chick embryos were harvested for various analyses including histological sections, Western immunoblotting, patch-clamp recordings of L-type voltage-gated calcium channels, and electroretinograms (ERGs).

### 2.3. Histology and Nissl Staining

Intact chicken eyes were submerged and fixed with Zamboni fixative (Newcomer Supply, Middleton, WI, USA) overnight at 4°C, followed by several washes in phosphate buffer (0.1 M PB, pH 7.4) and transferred to 10%, 20%, and 30% sucrose-PB solution for cryoprotection. The whole eye was sectioned at 12 *µ*m thickness and further processed for Nissl staining. After dehydration, coverslips were mounted, and images were observed and taken under a Zeiss microscope.

### 2.4. Plasma Glucose and Insulin Detections

Blood from E18 hearts was collected for blood glucose and insulin levels. A glucometer (Clarity Plus, Diagnostic Test Group, Boca Raton, FL, USA) was used to measure blood glucose levels. For insulin detection, blood samples (200–300 *µ*L per embryo) were stored overnight at 4°C to separate the serum from blood cells. Serum portions were collected by centrifugation (15 min; 2000 g). Serum insulin detection was detected using a chicken insulin ELISA kit (Cusabio, San Diego, CA, USA), which has higher sensitivities for chicken insulin than the regular insulin ELISA kits made for rodents or humans. The procedure was provided by the manufacturer. Results were analyzed against a standard curve.

### 2.5. Western Immunoblot Analysis

Retina tissue samples were collected and prepared as described previously [31]. Briefly, intact retinas were homogenized in a Tris lysis buffer including (in mM): 50 Tris, 1 EGTA, 150 NaCl, 1% Triton X-100, 1% *β*-mercaptoethanol, 50 NaF, and 1 Na_3_VO_4_; pH 7.5. Samples were separated on 10% sodium dodecyl sulfate-polyacrylamide gels by electrophoresis and transferred to nitrocellulose membranes. The primary antibodies used in this study were anti-glucose transporter 4 (anti-Glut4; Cell Signaling Technology, Danvers, MA, USA), anti-phosphorylated protein kinase B (AKT) at thr 308 (anti-pAKT-thr308; Cell Signaling Technology), anti-VGCC*α*1D (Alomone, Jerusalem, Israel), and anti-ERK (total ERK, used for loading control; Santa Cruz Biochemicals, Santa Cruz, CA, USA). Blots were visualized using appropriate secondary antibodies conjugated to horseradish peroxidase (Cell Signaling Technology) and an enhanced chemiluminescence (ECL) detection system (Pierce, Rockford, IL, USA). Relative protein expressions for all proteins involved in this study are reported as a ratio to total ERK. Band intensities were quantified by densitometry using Scion Image (NIH, Bethesda, MD, USA). All measurements were repeated at least 3 times.

### 2.6. Electroretinogram

At E20, chick embryos were anesthetized with an* in ovo* injection of 250 *µ*L tribromoethanol (Avertin) solution (12.5 mg/mL) into the amnion. The shell window was widened to expose the head, but the body was left in the egg to maintain body temperature. A small cut was made on the eye lid and the nictitating membrane to expose the cornea. The ground electrode was placed on top of the head, the reference electrode was placed under the skin in the cheek area, and the threaded recording electrode conjugated with a mini contact lens (OcuScience, Henderson, NV, USA) was placed on the surface of the cornea. A drop of Goniovisc (Hub pharmaceuticals, Rancho Cucamonga, CA) was applied on the surface of the cornea to keep it moist and to maintain proper contact between the cornea and the recording electrode. A portable ERG device (OcuScience) was used for the ERG recordings. Since we only focused on cone photoreceptor function, the photopic ERG was recorded. The chick embryo was adapted to 30,000 mcd*·*s/m^2^ background light for 10 min and exposed to 32 flashes (0.5 s interval) at 3000 mcd*·*s/m^2^ light intensity. The amplitudes and implicit time of a- and b-waves were recorded and analyzed using the ERGView 4.4 software (OcuScience). Throughout, egg temperature was maintained at 39°C using a custom-made digital temperature controller with a flexible heating tape (Briskheat, Columbus, OH, USA) surrounding the egg.

### 2.7. Dissociated Retinal Cultures and Electrophysiology

Retinas from E18 embryos were dissected, dissociated, and cultured on poly-D-lysine coated coverslips overnight in the presence of 20 ng/mL ciliary neurotrophic factor (CNTF, R&D Systems, Minneapolis, MN, USA) and 10% heat-inactivated horse serum as described previously [30, 32]. The cell culture incubator was maintained at 39°C and 5% CO_2_. Whole cell patch-clamp configuration of L-type voltage-gated calcium channel (L-VGCC) current recordings on cone photoreceptors was carried out using mechanically ruptured patches. The external solution was (in mM): 110 NaCl, 10 BaCl_2_, 0.4 MgCl_2_, 5.3 KCl, 20 TEA-Cl, 10 HEPES, and 5.6 glucose, pH 7.35 with NaOH. The pipette solution was (in mM): 135 Cs acetate, 10 CsCl, 1 NaCl, 2 MgCl_2_, 0.1 CaCl_2_, 1.1 EGTA, and 10 HEPES, pH 7.3 adjusted with CsOH. Recordings were made only from cells with elongated cell bodies with one or more prominent oil droplets (hallmark of avian cone photoreceptors) [27, 28, 33]. Currents were recorded at room temperature (23°C) using an Axopatch 200B (Molecular Devices, Union City, CA, USA) or A-M 2400 amplifier (A-M Systems Inc., Carlsborg, WA, USA). Signals were low-pass filtered at 2 kHz and digitized at 5 kHz with Digidata 1440A interface and pCLAMP 10.0 software (Molecular Devices). Electrode capacitance was compensated after gigaohm seals were formed. Cells were held at −80 mV, and current-voltage (*I*-*V*) relations were elicited from the holding potential in 200 ms steps (5 s between steps) to test potentials over a range of −80 to +20 mV in 10 mV increments. The maximal currents were obtained when the steps depolarized to 0~+10 mV. The membrane capacitance, series resistance, and input resistance of the recorded photoreceptors were measured by applying a 5 mV (100 ms) depolarizing voltage step from a the holding potential. Cells with an input resistance smaller than 1 GΩ were discarded. The membrane capacitance reading was used as the value for whole cell capacitance. The current densities (pA/pF) were obtained by dividing current amplitudes by membrane capacitances. Leak currents were subtracted manually after data acquisition.

### 2.8. Statistics

All of the data are presented as mean ± s.e. (standard error). Student's* t*-test was used to compare the control and STZ-induced diabetic groups. One-way ANOVA followed by Tukey's* post hoc* test for unbalanced* n* was used for comparisons of multiple groups. Throughout, ∗*P* < 0.05 was regarded as significant.

## 3. Results and Discussion

### 3.1. Successful Induction of Diabetes in Chicken Embryos

**Figure 1:**
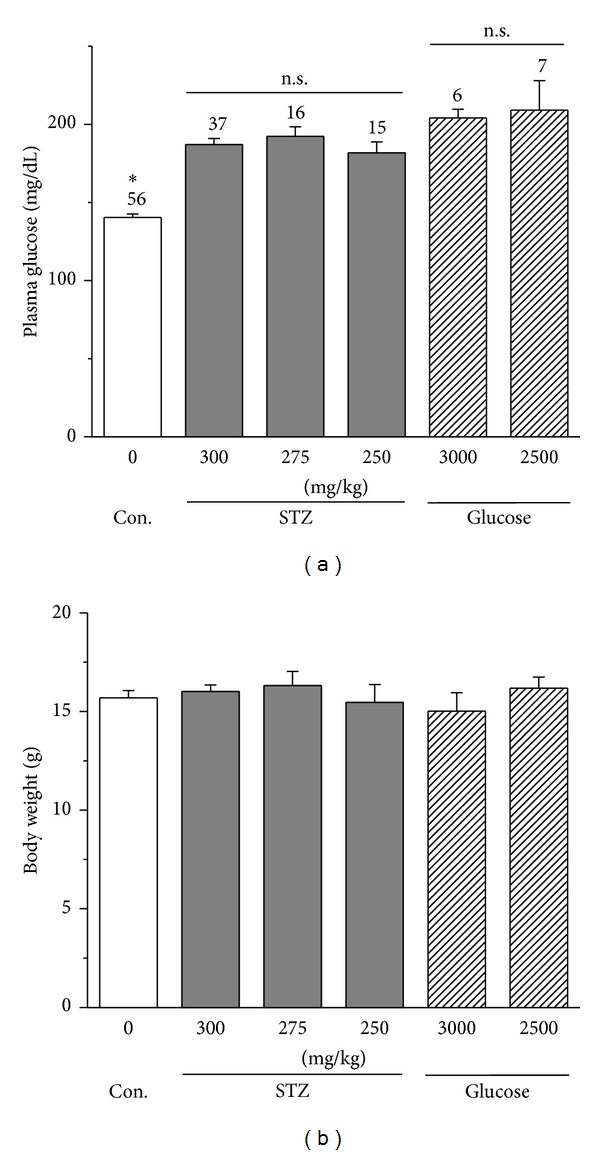
STZ- or high glucose-injected embryos have elevated plasma glucose levels without changes in body weights. (a) At E12, embryos were injected with either STZ (300, 275, or 250), high glucose (3,000 or 2,500) mg per kg of egg weight (mg/kg), or vehicle (control). All STZ- and high glucose- injected embryos at E18 had significantly higher plasma glucose levels compared to the control (CON). (b) There is no statistical difference in body weight among all groups. *P < 0.05. n.s.: no statistical difference.

We first had to determine the best way to induce diabetes in chicken embryos through* in ovo* injections of STZ or high concentrations of glucose (high glucose). We originally injected STZ into the embryos directly, but the toxicity of STZ persistently killed the embryos. After testing several injection sites external to the embryo (amnion layer, allantois, albumen, and yolk sac), we discovered that injecting the amnion layer at embryonic day 12 (E12) gave us the best outcome, in which the survival rate was higher than direct injections into the embryos (Table 1), as well as successful induction of hyperglycemia by E18 (Figure 1(a)). We tested three different doses of STZ (300, 275, and 250 mg per Kg of egg weight [mg/kg-egg]) and two for glucose (3,000 and 2,500 mg/kg-egg). One week after injections, both STZ- and high glucose-injected embryos displayed a significant increase of plasma glucose levels (Figure 1(a)), and, thereafter, we used hyperglycemia as an index of successfully induced diabetes in chicken embryos. Even though the high glucose injections elicited higher levels of hyperglycemia (Figure 1(a)), they also caused a higher overall death rate compared to the STZ injections (Table 1). However, there was no significant change in embryo body weight after STZ or high glucose injection compared to the controls (injected with the vehicle; Figure 1(b)). If we had observed any major body weight changes after 1 week of STZ or high glucose injections, it might reflect that STZ or high glucose injections had compromised the global development of the chicken embryos.

**Figure 2:**
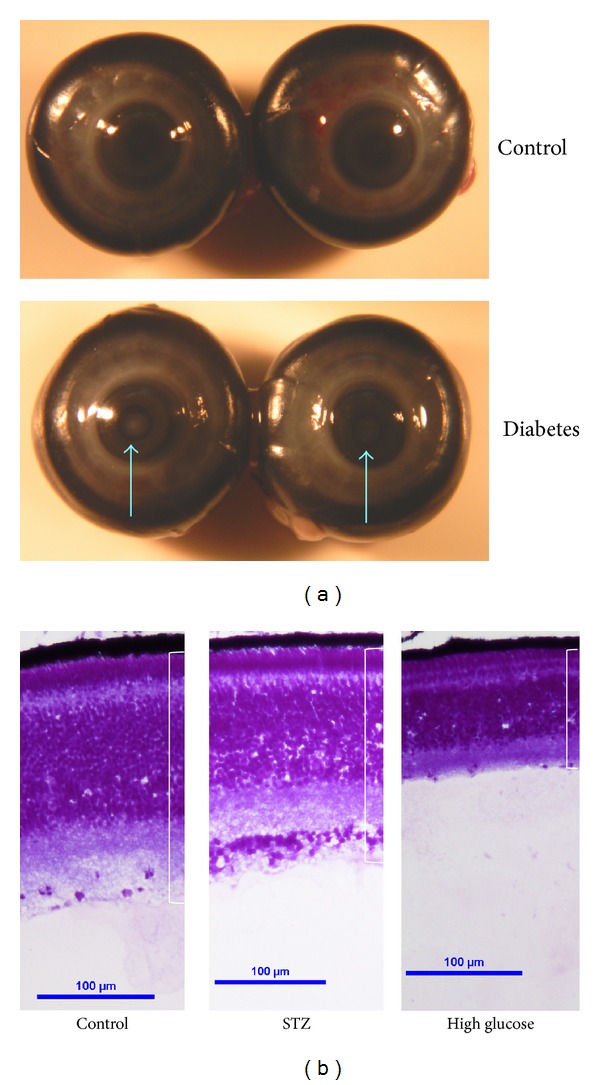
There are morphological changes in STZ- and high glucose-induced diabetic eyes. (a) Cataracts (arrows) occur in STZ- or high glucose-induced diabetic embryos at E18. (b) The retinas of STZ- and high glucose-induced diabetic eyes are thinner than the control eyes of the same age (E18).

In addition, we also observed cataracts in STZ- and high glucose-injected embryos (Table 1 and Figure 2(a)). In the USA, about 24% of patients with early onset diabetes (mainly type I) suffer from cataracts [34]. Similarly, cataracts occur in STZ-induced diabetic rats [35]. STZ- or high glucose-induced cataracts to varying degrees but always at a significantly higher rate compared to those that arise spontaneously in normal chicken embryos. While we observed cataracts in both STZ and hyperglycemia-induced diabetic eyes, STZ injections apparently induced cataracts at a higher rate than hyperglycemia-induced diabetic eyes with injections of STZ at 300 mg/kg-egg inducing the highest rate of cataract (Table 1). We cannot rule out the possibility of chemical damage by STZ in chicken embryos. Both STZ and high glucose injections caused retinal degeneration, as shown in Figure 2(b) where overall retina thickness was decreased compared to the control, but the degree of change in retinal thickness varied. Therefore, the morphological data provided evidence that injections of STZ or high glucose were able to induce diabetic retinas in chicken embryos. One concern with the high glucose treatment was the apparent dramatic difference in retinal thickness from the control and STZ-injected groups. It could be due to the effect of high glucose during retinal development, even though the survival rate of the 2500 mg/kg glucose treatment seemed to be acceptable (Table 1). Taking all factors into consideration, including the survival of embryos, the occurrence of cataract, general retinal morphology, and hyperglycemic index, we determined that the best strategy to induce diabetes in chicken embryos was a single injection of STZ at 300 mg/kg-egg into the amnion layer. Hence, we focused on the characterizations of STZ-induced diabetic chicken embryos in the following analyses.

### 3.2. Molecular Markers of STZ-Induced Diabetic Chicken Embryos

**Figure 3:**
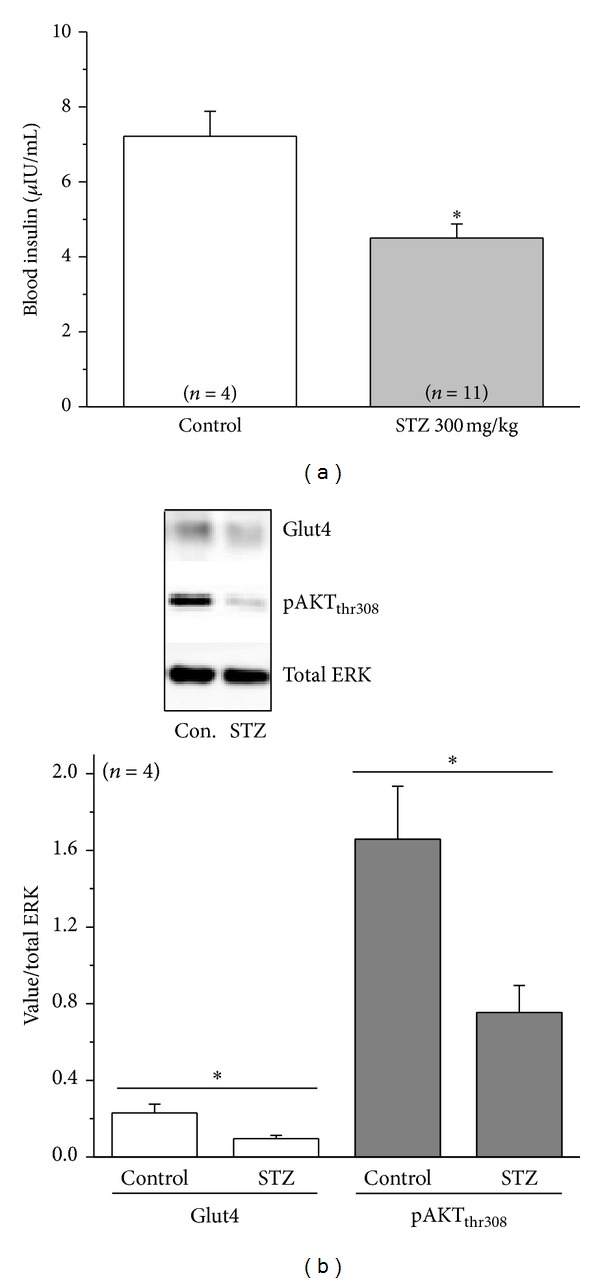
STZ-injected embryos display changes in biomarkers that are characteristic to diabetes. (a) Blood insulin from STZ-injected embryos (E18) is significantly lower than the control (injected with vehicle). (b) The protein levels of glucose transporter 4 (Glut4) and phosphorylated AKT at thr308 (pAKT_thr308_) are significantly lower in the STZ-induced diabetic retinas compared to the control.

Streptozotocin is known to deplete whole body insulin and induce diabetes in animals by destroying pancreatic beta-cells [36–38]. We used an ELISA-based assay kit to detect blood insulin levels (Figure 3(a)). Even though the embryonic pancreas is not fully developed, insulin-positive pancreatic cells are present in chicken embryos from E10 [26]. At E18, blood insulin level was low in the control group but was significantly decreased in STZ-injected embryos (Figure 3(a)).

We next examined several molecular markers that are linked to insulin signaling and changed under diabetic conditions. We found that one week after STZ injections both Glut4, the dominant glucose transporter in the retina, and phosphorylated AKT at thr308 (pAKT_thr308_) were significantly decreased in the E18 diabetic retina (Figure 3(b)), similar to previous reports in STZ-induced diabetic rats [39–45]. Hence, results from these molecular markers provide evidence that STZ injections were able to induce diabetic retinas in chicken embryos.

### 3.3. Physiological Evidence for STZ-Induced Diabetic Retina in Chicken Embryos

**Figure 4:**
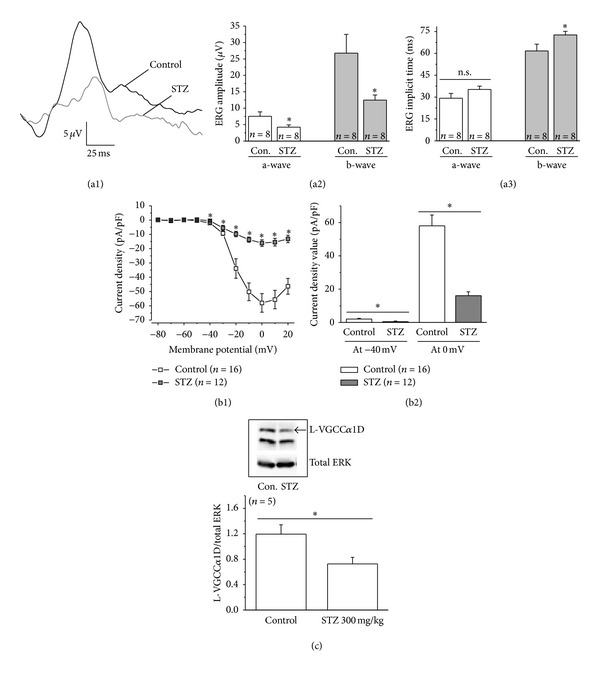
The STZ-induced diabetic retina exhibits changed physiology. (a1-a3) ERGs were recorded from E20 embryos. (a1) Representative ERG waveforms from a control and a STZ-injected embryo. (a2) The amplitudes of a- and b-waves from STZ-injected embryos are significantly lower than the ones recorded from controls. (a3) The implicit time of both a- and b-waves from STZ-injected embryos are delayed, but there is a significant increase of the b-wave implicit time from STZ-injected embryos compared to the control. (b1-b2) Cone photoreceptors from control and STZ-injected embryonic retinas were dissociated and cultured at E18. The next day, patch-clamp recordings of L-VGCCs were carried out. (b1) The average data of voltage (mV) current density (pA/pF) relationship from the control and STZ-diabetic photoreceptors are shown. (b2) At both −40 mV and 0 mV, the L-VGCC current densities are significantly lower from STZ-diabetic photoreceptors compared to the control. (c) The STZ-injected diabetic retina has a significant decrease in the protein level of the L-VGCC*α*1D subunit compared to the control. Total ERK serves as a loading control. *P < 0.05.

In STZ-induced diabetic rats, the amplitudes of ERG a- and b-waves are decreased, and the implicit times for both are increased compared to the control [35, 46]. In humans, early diabetic patients without DR have delayed ERG a-wave implicit time in cone responses [12, 13, 16, 47, 48]. We found that 9 days after injections (ERGs recorded at E20), STZ-injected embryos had significant decreases in the amplitudes of both photopic ERG a- and b-waves but with a significant increase in the b-wave implicit time compared to the control (Figure 4(a)). We performed ERG recordings on 13 STZ-injected embryos, but 5 completely had no currents, while all controls (*n* = 8) displayed ERGs (Figure 4(a)). Hence, we only included the 8 STZ-injected embryos that had reputable ERG waveforms in our statistical comparison.

Since L-type voltage-gated calcium channels (L-VGCCs) are responsible for neurotransmitter release from photoreceptors and bipolar cells [49], we cultured photoreceptors from E18 embryos and used patch-clamp recordings to assess whether the changes in ERGs correspond to any alteration in L-VGCCs. We found that, starting at −40 mV step voltage, photoreceptors obtained from STZ-injected embryos had significant decreases in L-VGCC current densities (Figure 4(b)), and Western blot analyses showed that these embryos had a significant decrease in the protein level of the L-VGCC*α*1D subunit (Figure 4(c)), the major L-VGCC*α*1 subunit in cone photoreceptors [50–53]. Therefore, physiological data from both ERGs and patch-clamp recordings display our ability to induce diabetic retinal degeneration in chicken embryos by STZ injections.

## 4. Conclusions

In humans, cone photoreceptors are compromised in early diabetes prior to DR, and, currently, there is no treatment or cure to revert degenerated photoreceptors back to prediabetic states. Current animal models for DR research mostly use rod-dominant animals, which prompted us to establish a cone-dominant animal model to address cone dysfunction in prediabetic or early diabetic conditions. Our results indicated that STZ injections into the amnion layer* in ovo* induced diabetic pathology in chicken embryos. We compared the death and cataract rates between STZ- and high glucose-induced diabetes (Table 1) and determined that a single injection of STZ at 300 mg/kg-egg weight induced the best result with a lower death rate but higher incidence of cataracts. We then further looked for STZ-induced changes in biomarkers and retinal physiology. As cataracts are a hallmark of early onset type I diabetes in humans, our model might be suitable for type I diabetes research. In agreement with the rodent models, Glut4 and pAKT were decreased in the diabetic retinas. Intracellular phosphoinositol 3 kinase (PI3K) AKT signaling is important for cell growth, metabolism, and survival [54] and is essential for insulin-mediated glucose uptake and maintaining normal glycemia [55–57]. Insulin activates intracellular Glut trafficking from the cytosol to the plasma membrane, mediated by PI3K-AKT signaling, and insertion of Glut into the plasma membrane facilitates cellular glucose intake [58–61]. Glucose is the major energy source for neurons in the retina and brain [62–64]; thus, PI3K-AKT mediated Glut trafficking is essential for neuron survival. The dominant Glut present in the retina is the type 4 (Glut4). The decreases of Glut4 and pAKT in the STZ-diabetic chicken retina confirmed that STZ injections into chicken embryos successfully induced pathological conditions in early diabetic retina.

We further established a stable ERG recording method from anesthetized chick embryos with body temperature maintained at 39°C. We performed all ERG recordings from E20 instead of E18 because E20 embryos gave us more reliable ERG recordings. Since our purpose in this study was to establish a cone-dominant diabetic retinopathy animal model, we only performed ERG recordings with the light-adapted photopic responses. We found that both a- and b-wave amplitudes were significantly decreased in STZ-injected embryos. The implicit times of both a- and b-wave from STZ-injected embryos were also delayed, with a significant increase on the b-wave implicit time compared to the control. Our ERG results echo those from human patients where cone pathway responses are weakened in early diabetic patients [9, 10, 13, 46, 47]. Since L-VGCCs are responsible for synaptic transmission from photoreceptors, the ERG results were consistent with our finding that cones cultured from STZ-injected embryos had significantly lower amplitudes of L-VGCC currents, which coincides with the significant decrease of L-VGCC*α*1D expression in the STZ-injected retina compared to the control. Our patch-clamp recording of L-VGCCs from cultured cone photoreceptors was the first to indicate that diabetic conditions might severely impact the expression and plasma insertion of L-VGCCs. We previously showed that PI3K-AKT is responsible for L-VGCC*α*1 subunit trafficking and insertion into the plasma membrane in cone photoreceptors [32]. Hence, the decrease of phosphorylation/activity of PI3K-AKT [39, 58] by diabetic conditions might be one of the reasons leading to the decrease of L-VGCCs.

One potential caveat of this study is that while STZ injections induced a significantly higher blood glucose level in STZ-embryos compared to controls, the increase of the plasma glucose level is not as high as what is observed in the STZ-induced diabetic rodents (from ~150 mg/dL of controls to ~250–400 mg/dL after STZ injections). We postulate that the blood glucose level increased at a much higher rate in rodents because animals used in the mammalian STZ-animal models already consume food with insulin- mediated blood glucose regulation by the time of STZ injections. But chicken embryos do not “eat,” and the pancreas has not yet fully developed, so naturally, chicken embryos might not display a large surge in blood glucose levels 1 week after STZ injections. While STZ injections into the amnion layer induced diabetic pathology in the chick embryonic eyes (cataract and other pathophysiology of the retina), which is comparable to the STZ-induced diabetic rodent models, we cannot completely rule out the possibility that observed retinal pathology in STZ-injected chicken embryos could potentially contribute to the detrimental effects of developing retinas. Since STZ injections often fail to induce diabetes in adult birds because of species-dependence in STZ-sensitivity [23, 24], we hence tried to induce diabetic conditions in chicken embryos. Future research on the hatched/young adult STZ-chickens will be needed to ensure the establishment of diabetes in adult STZ-chickens, which will complement the existing animal models for diabetic research and will further assist in treating or preventing cone degeneration in human diabetic patients in the future.

## Figures and Tables

**Table 1:** The numbers and rates of death and cataract after various STZ or high-glucose injections. Various doses of STZ or high-glucose were injected into the chick embryos at E11, and the data were taken at E18.

Group	Total (number)	Survival (number)	Death (number)	Cataract (number)	Death rate % (death/total)	Cataract rate % (Cataract/Survival)
Control	61	56	5	3	8%	5.4%
STZ 300 mg/kg-egg	46	37	9	32	19.6%	86.4%
STZ 275 mg/kg-egg	21	16	5	9	23.4%	56.2%
STZ 250 mg/kg-egg	22	15	7	5	31.8%	30%
Glucose 3000 mg/kg-egg	23	6	17	3	74%	50%
Glucose 2500 mg/kg-egg	9	7	2	3	22.2%	41.8%
